# Development of ‘Redox Arrays’ for identifying novel glutathionylated proteins in the secretome

**DOI:** 10.1038/srep14630

**Published:** 2015-09-29

**Authors:** Lisa Mullen, Miles Seavill, Raneem Hammouz, Barbara Bottazzi, Philippe Chan, David Vaudry, Pietro Ghezzi

**Affiliations:** 1 Brighton and Sussex Medical School, Trafford Centre for Medical Research, Falmer, Brighton BN1 9RY UK; 2 Humanitas Clinical & Research Center Via Manzoni, 113, 20089 Rozzano, Milano, Italy; 3 Platform in Proteomics PISSARO, Institute for Research and Innovation in Biomedicine, University of Rouen,76821 Mont-Saint-Aignan, France

## Abstract

Proteomics techniques for analysing the redox status of individual proteins in complex mixtures tend to identify the same proteins due to their high abundance. We describe here an array-based technique to identify proteins undergoing glutathionylation and apply it to the secretome and the proteome of human monocytic cells. The method is based on incorporation of biotinylated glutathione (GSH) into proteins, which can then be identified following binding to a 1000-protein antibody array. We thus identify 38 secreted and 55 intracellular glutathionylated proteins, most of which are novel candidates for glutathionylation. Two of the proteins identified in these experiments, IL-1 sRII and Lyn, were then confirmed to be susceptible to glutathionylation. Comparison of the redox array with conventional proteomic methods confirmed that the redox array is much more sensitive, and can be performed using more than 100-fold less protein than is required for methods based on mass spectrometry. The identification of novel targets of glutathionylation, particularly in the secretome where the protein concentration is much lower, shows that redox arrays can overcome some of the limitations of established redox proteomics techniques.

Glutathione (GSH) has a key signalling role in redox regulation^1^,^2^,^3^. A molecular mechanism for the regulatory action of GSH is protein glutathionylation, a post-translational modification in which glutathione (GSH) forms a disulphide bond with a cysteine of a protein. It is now well accepted that glutathionylation, which is a reversible process, plays important roles in the redox regulation of protein activity and cell signalling^4^,^5^,^6^,^7^,^8^.

Key signalling proteins in infection and immunity that can be regulated by glutathionylation include p50^9^, STAT3^10^, and HIV protease^11^. We and others previously showed that protein glutathionylation can be regulated by macrophage activators^12^ and HIV infection^13^. Interestingly, a number of intracellular proteins that can be released and act as endogenous inflammatory signalling molecules (also known as ‘damage-associated molecular patterns’) can undergo glutathionylation, including high mobility group box 1 protein (HMGB-1)^14^, proteins of the S100 family^15^, galectin-1^16^, peroxiredoxin-2 (Prdx2)^17^ and heat-shock protein 70 (HSP70)^18^. Redox proteomics methods for identifying glutathionylated proteins have been developed, including labelling of GSH either by ^35^S^16^ or biotinylation^17^,^19^,^20^. However, these approaches suffer from a common limitation of proteomic methods, where the presence of proteins in high-abundance make the identification of ones present in low-abundance very difficult. For this reason, abundant proteins such as Prdxs, HSPs, enolase-1 and keratin appear again and again in proteomics experiments^21^. Glutathionylation could potentially affect many biologically important secreted and intracellular proteins that are present in very low concentrations and consequently difficult to identify with the usual proteomics technologies^22^,^23^. We describe here the development of a ‘redox array’ technology which aims to identify glutathionylated proteins irrespective of their relative abundance. The method is based on a similar methodology to that used in our previous study using BioGEE to label the proteins undergoing glutathionylation^17^. The sample is then applied to a commercially available antibody array for 1000 human proteins and glutathionylated proteins visualized with streptavidin-peroxidase.

## Results

### Identification of secreted glutathionylated proteins from LPS-treated monocytic cells

**Figure 1:**
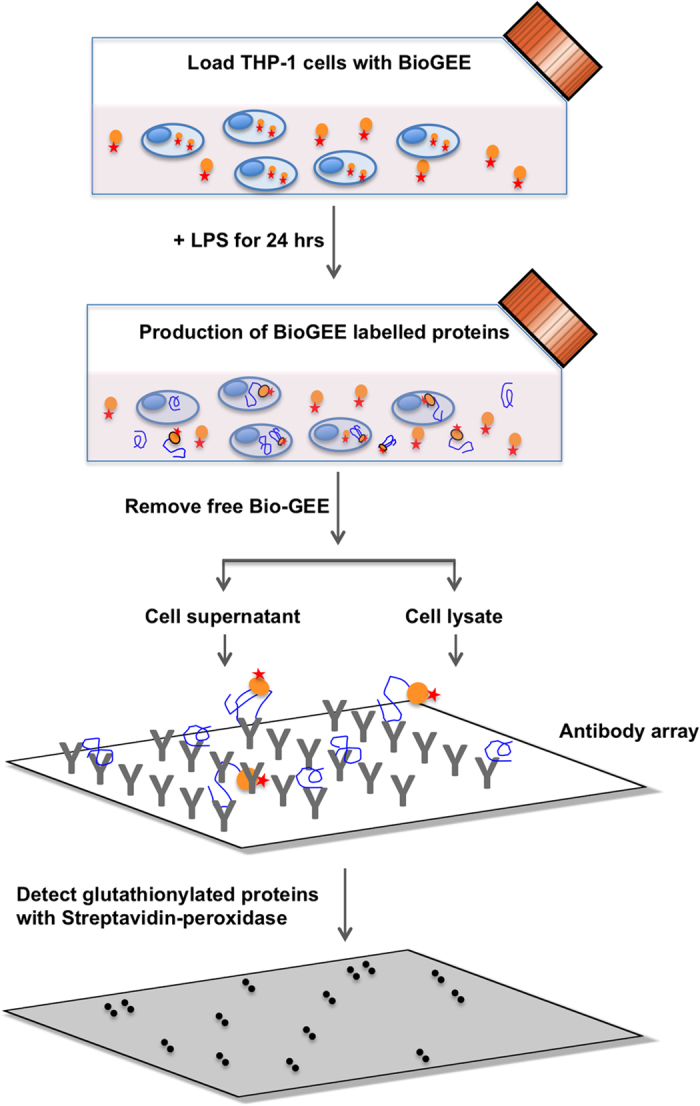
Schematic representation of the experimental procedure used for the redox arrays. THP-1 cells were incubated with cell-permeable BioGEE to label the intracellular pool of proteins. After 24 hour incubation with LPS to allow secretion of proteins, cell conditioned medium and cell lysates were collected. After removal of free BioGEE, proteins were applied to the antibody membrane-based array. Biotin-labelled proteins were detected by the addition of streptavidin-HRP, followed by chemiluminescent detection.

**Figure 2:**
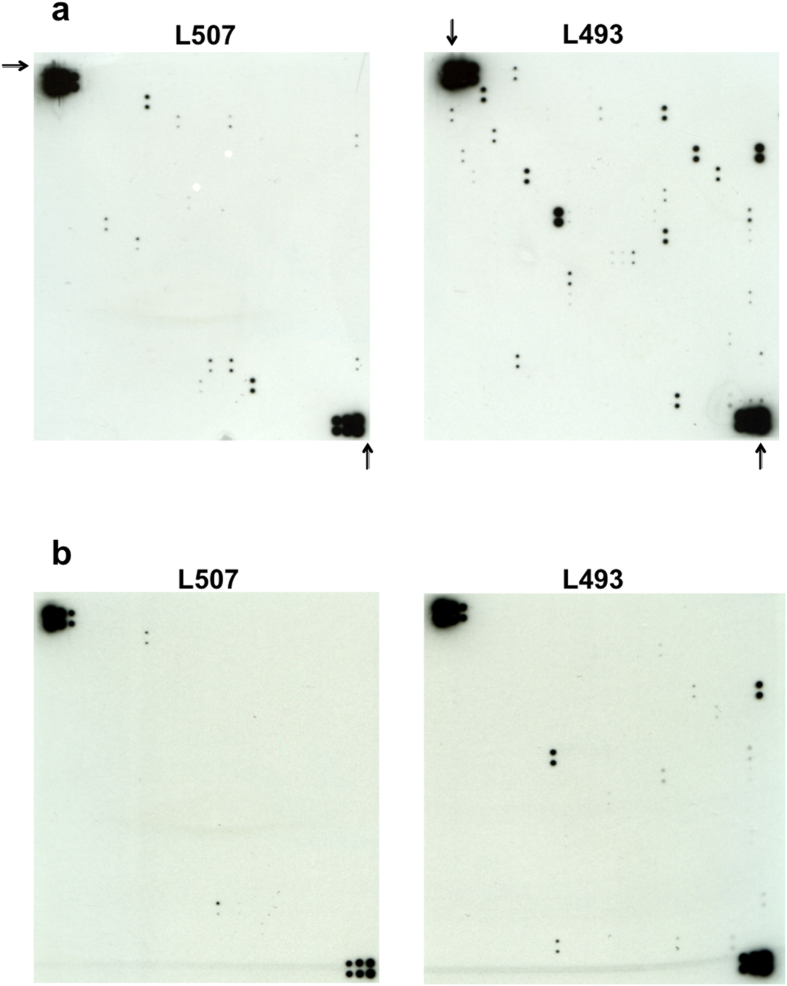
L1000 antibody arrays (composed of L507 on the left and L493 on the right) identify a number of glutathionylated proteins secreted from LPS-treated THP-1 cells. (**a**) Conditioned media from LPS-treated BioGEE-loaded THP-1 cells were applied to the array. Streptavidin-HRP was used to detect the presence of bound biotinylated proteins. Triplicate positive control spots are indicated by arrows. (**b**) A second aliquot of the same sample was reduced with DTT and applied to a second array.

Human monocytic THP-1 cells were pre-loaded with BioGEE, and then stimulated with lipopolysaccharide (LPS). Cells were treated with LPS as LPS increases protein transcription, translation and secretion, thereby increasing the concentration of secreted proteins. LPS also acts as an inflammatory stimulus in these cells, a condition which has previously been shown to increase protein glutathionylation and may thus enable the identification of novel targets for glutathionylation as part of the inflammatory response. After 24 h, supernatants were collected and free BioGEE removed using desalting columns before applying to a L1000 antibody array. Bound biotinylated proteins were then identified using streptavidin-HRP and subsequent detection by ECL (Fig. 1). Antibody spots on the arrays were considered positive if they were visible in duplicate. The number of positive spots was determined at an exposure of 5 min for all membranes. Longer exposures resulted in increasing the background without increasing the number of positive spots. The intensity of the signal was not considered when determining which spots were positive, as this could depend on the number of cysteine residues undergoing glutathionylation and the absolute amount of the protein. To confirm the specificity of BioGEE labelling of these proteins, a second aliquot of the THP-1 supernatant was reduced with DTT to release the BioGEE from the protein by reducing the disulphide bond and applied to a second L1000 array (Fig. 2). We could thus identify a total of 38 potential targets of glutathionylation as a number of proteins, 17 of the 55 detected, were still found on the array even after removal of the BioGEE label by DTT treatment. The proteins identified are listed in Table 1.

### Identification of intracellular glutathionylated proteins in LPS-treated monocytic cells

For the analysis of cell lysates, the protein concentration was adjusted to 0.3 mg/mL and applied to the array and processed as described above.

To identify false positives, a second aliquot of the sample was treated with DTT as described above. A number of proteins, 19 in total, were still detected on the array even after reduction (Table 2) and so represent false positives, leaving a total of 55 proteins identified as targets of glutathionylation. A number of proteins previously shown to be glutathionylated were also identified here as shown in Table 2. Of note, IL-11 has no cysteines but its intracellular precursor has two^24^, so it is interesting that it was only identified intracellularly and not in the secretome. This also suggests that the separation of intracellular and secreted proteins was successful, otherwise there would be a positive signal for the IL-11 antibody on the array used for detection of glutathionylated secreted proteins.

### Identification of glutathionylated proteins in untreated monocytic cells

Finally we performed similar experiments in the absence of LPS. The number and identity of secreted glutathionylated proteins were very similar, but not identical, between LPS-treated and control cells (Table 3). Cell lysates were also prepared from cells without LPS treatment to determine if any proteins were glutathionylated specifically in response to LPS. The number and identity of glutathionylated proteins were identical in lysates from LPS-treated and unstimulated cells (data not shown).

### Validation of novel glutathionylated proteins in THP-1 cells

**Figure 3:**
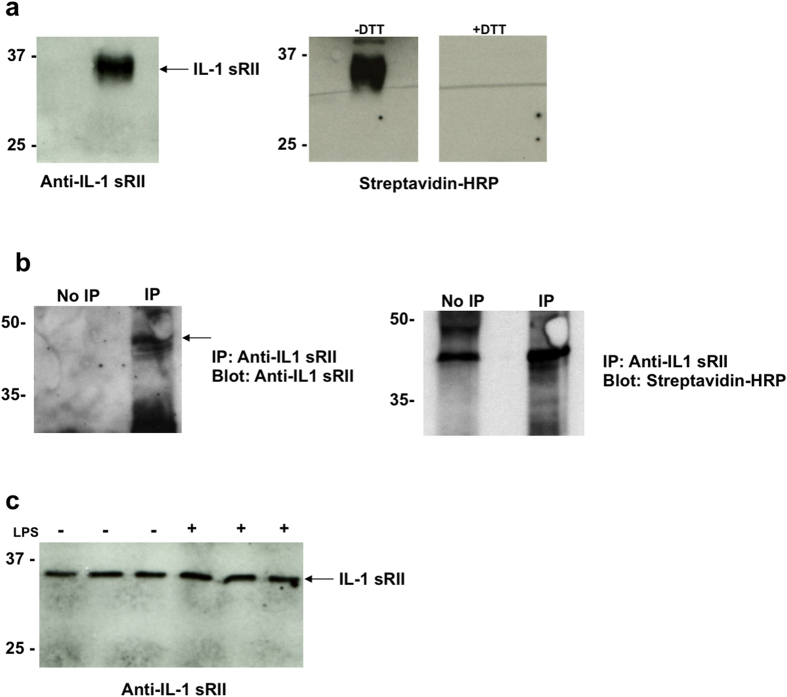
Recombinant IL-1 sRII is glutathionylated *in vitro* and in THP-1 cells. (**a**) Western blotting of recombinant IL-1 sRII treated with BioGEE *in vitro* probed with anti-IL-1 sRII antibody (left panel) or streptavidin-HRP (right panel). The signal from the streptavidin-labelled protein is lost upon treatment with DTT. (**b**) Immunoprecipitation of IL-1 sRII from cell culture media of untreated THP-1 cells preincubated with BioGEE. Biotin labelling of the IL-1 sRII was confirmed by stripping of the membrane and re-probing with streptavidin-HRP. (**c**) Release of IL-1 sRII from THP-1 cells in response to treatment with LPS.

**Figure 4:**
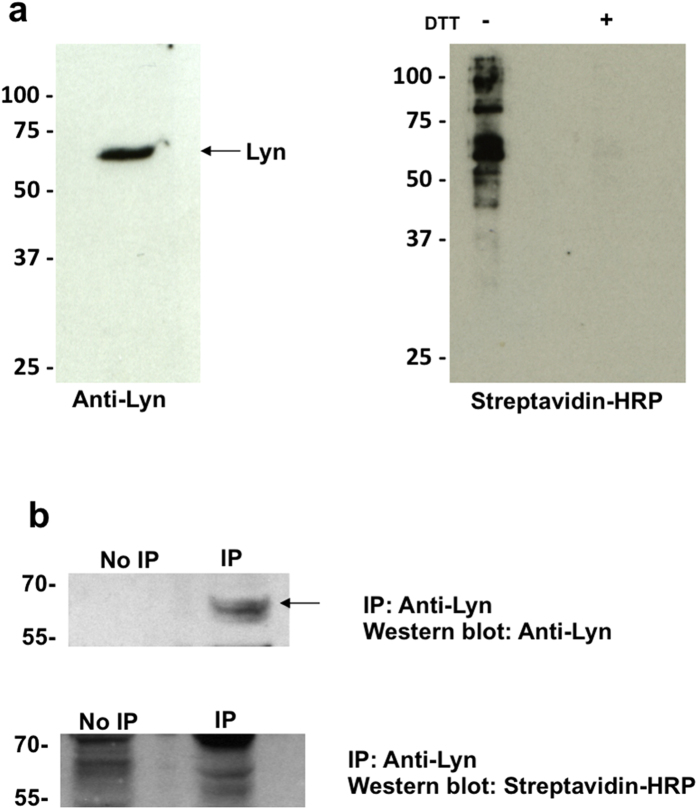
Recombinant Lyn is glutathionylated *in vitro* and in THP-1 cells. (**a**) Western blotting of recombinant Lyn treated with BioGEE *in vitro* probed with anti-Lyn antibody (left panel) or streptavidin-HRP (right panel). The signal from the streptavidin-labelled protein is lost upon treatment with DTT. (**b**) Immunoprecipitation of Lyn from cell lysates of untreated THP-1 cells preincubated with BioGEE. Biotin labelling of Lyn was confirmed by stripping of the membrane and re-probing with streptavidin-HRP.

To validate this method of identifying glutathionylated proteins, we selected one intracellular protein, Lyn, and one secreted protein, IL-1s RII, that had never been shown before to be glutathionylated. To confirm that these proteins were susceptible to glutathionylation, we treated the recombinant protein with BioGEE *in vitro* and analysed the extent of BioGEE labelling by Western blot using streptavidin-HRP. Both IL-1 sRII (Fig. 3a) and Lyn (Fig. 4a) were heavily labelled with BioGEE and the signal was lost in each case upon treatment with DTT, which confirmed the specificity of the signal as due to a mixed disulfide. The identity of each protein was also confirmed by probing with antibodies specific for IL-1 sRII and Lyn respectively, but due to the strength of the streptavidin-HRP signal, this was done on separate membranes where more protein was loaded per well for detection with specific antibodies. To confirm that only proteins susceptible to glutathionylation were detected using this method, TNF was used as a negative control as it was not detected in any of the redox arrays, but does contain 4 cysteines. Recombinant TNF was not labelled with BioGEE in the presence of diamide in this *in vitro* system as shown in supplementary Fig. S1.

Having shown that both IL-1 sRII and Lyn were susceptible to glutathionylation *in vitro*, we next wanted to confirm that these proteins were glutathionylated under physiological cellular conditions. IL-1 sRII or Lyn was immunoprecipitated from conditioned medium or cell lysates of THP-1 cells that were pre-incubated with BioGEE. Biotin labelling of these proteins was demonstrated by Western blotting using streptavidin-HRP (Figs 3b and 4b), confirming the glutathionylation of these proteins in THP-1 cells.

IL-1 sRII was identified in the redox array of the secreted proteins from both unstimulated and LPS-treated cells (Tables 1 and 3), but only in one replicate for each experimental condition. Having confirmed the glutathionylation of this protein both *in vitro* and in THP-1 cells, we hypothesized that it was not detected in all replicates of the array due to the low concentration present in the cell supernatants. Such a hypothesis was supported by the fact that IL-1 sRII could not be detected by Western blotting without immunoprecipitation (Fig. 3b). Precipitation of protein from THP-1 cell conditioned media, followed by Western blotting analysis showed similar amounts of IL-1sRII in the supernatants of unstimulated and LPS-treated cells (Fig. 3c), but, again, could only be detected after concentration of the protein by acetone precipitation prior to SDS-PAGE and Western blotting. Finally, the absolute concentration of IL-1 sRII in the supernatants of THP-1 cells was measured by ELISA. The concentration of IL-1 sRII was 30 ± 0.04 pg/mL in the supernatants of both LPS-treated and untreated cells.

### Identification of BioGEE labelled proteins by mass spectrometry

The redox arrays were clearly very sensitive in detecting biotinylated proteins. However, we wanted to compare the array method to more conventional methods of identifying glutathionylated proteins using BioGEE, for example, by mass spectrometry following affinity purification on streptavidin beads^17^,^25^. To allow a direct comparison to the redox array described here, aliquots of the desalted cell supernatants from THP-1 cells preloaded with BioGEE ± LPS were applied to streptavidin agarose using the same volume and concentration of desalted supernatant as that applied to the redox arrays. However, no proteins could be visualised by Coomassie staining of SDS-PAGE gels, nor were any proteins identified by mass spectrometry (data not shown). This was most likely due to the low quantity of proteins recovered from the streptavidin beads. Even after concentration of these samples eluted from the streptavidin beads, the mass spectrometry profile showed only low intensity peptides and high background noise. These experiments were then repeated using 120-fold more input protein for the binding to streptavidin agarose and subsequent elution and separation by SDS-PAGE. A number of intensely stained bands were observed by Coomassie staining of SDS-PAGE gels and a number of proteins were identified by mass spectrometry after tryptic digestion of gel bands as shown in Supplementary Table S1. A cut-off of 2 or more unique peptides and a score of 20 or higher were used to identify glutathionylated proteins in these experiments. A number of proteins are known to bind non-specifically to agarose beads and so tend to be identified in numerous proteomics screening experiments^26^. Therefore, those proteins that have previously been identified as a result of non-specific binding to agarose beads^25^,^26^ were removed from the list of identified proteins in supplemental Table S1. Only 4 proteins identified by mass spectrometry were present on the array, but not identified as glutathionylated in the array experiments described above: complement C3, PEDF, thrombospondin and vitamin D binding protein.

## Discussion

We describe and validate here a novel method for identifying both intracellular and secreted glutathionylated proteins using a ‘redox array’. A total of 74 and 55, from a total repertoire of 1000, of the antibody spots were positive on initial screening using cell lysates or conditioned media respectively. Treatment of the proteins with DTT prior to application to the array allowed us to distinguish between those proteins where BioGEE had been incorporated into the protein via a non-disulphide mechanism (possibly due to interaction with biotin) from those that formed disulphide bonds with the GSH in BioGEE and hence were more likely to represent potential targets of glutathionylation. Using this approach 38 secreted and 55 intracellular potential targets of glutathionylation were identified. Fewer glutathionylated proteins were found in the secreted samples, compared to the cell lysates as expected, given the different relative amounts of each protein present in these samples. These observations are also in agreement with the fact that intracellular proteins normally contain more free cysteines than secreted proteins^27^, which are richer in disulfide bonds. GSH is present mainly intracellularly, which also favours formation of mixed disulfides of intracellular proteins.

The importance of repeating the array with the same sample treated with a reducing agent is critical to identify false positives. A number of spots were positive even after treating the protein sample with DTT so that the signal could not have been due to the presence of the biotin label attached via a disulfide bond. Although we considered those as non-specific binding, it should be noted that GSH can bind proteins via stable, non-reducible, thioethers, and these could account for some of these positive spots^28^.

To further validate this method we confirmed that both Lyn and IL-1sRII are susceptible to glutathionylation *in vitro* and provide the first evidence that these proteins undergo glutathionylation in THP-1 cells. Intriguingly, Lyn has previously been identified as a redox sensor that mediates neutrophil recruitment *in vivo* using a zebrafish larvae model system^29^. This same study also demonstrated by BIAM labelling that Lyn has multiple cysteines that can be oxidized. It appears that Lyn is glutathionylated in THP-1 cells even in the absence of LPS stimulation, at least on one or more of the 8 cysteine residues present.

Glutathionylated IL-1 sRII was identified in the redox secretome of unstimulated and LPS-treated THP-1 cells. We validated the potential for glutathionylation of this protein *in vitro* and also showed that IL-1 sRII is secreted from both unstimulated and LPS-treated THP-1 cells. This is the first evidence, to our knowledge, that this protein is glutathionylated and this is most likely because of the very low concentration in the secretome which is below the limit of detection for most proteomic methods. The biological consequences of glutathionylation for both Lyn and IL-1 sRII are currently under investigation.

The fact that few differences were found between the LPS-treated and untreated samples was somewhat surprising, as LPS treatment has previously been shown to increase glutathionylation of a number of proteins in mouse macrophages^25^. However, the ability to detect LPS-induced glutathionylated proteins by the redox array method is restricted by the number and identity of the antibodies included on the array. Of note, none of the 5 previously identified targets of glutathionylation (peroxiredoxin 1, peroxiredoxin 2, thioredoxin, profilin and vimentin) were present on the antibody array used here. Therefore, it may be that the proteins most responsive to glutathionylation in response to LPS were simply not present on the array.

Unsurprisingly, many more proteins were identified by mass spectrometry than in the array, given that the array was restricted to a total possible pool of 1000 proteins. The fact that the list of proteins identified by the redox array was so different from the list identified in the mass spectrometry experiments was intriguing. Indeed, this would indicate that there are significant differences in the detection capabilities of these two methods and this should be considered when deciding on an appropriate methodology for particular biological applications. It is likely that these differences in the two methods arise from differences in sensitivity, given that our first mass spec experiment failed to identify any proteins when conducted using the same amount of input material as used for the redox arrays. This hypothesis is further supported by the fact that we were able to identify glutathionylated IL-1 sRII by redox array even when the concentration in cell conditioned media was only 30 pg/mL. The fact that this protein was not identified in the mass spectrometry experiments was most likely due to the low relative abundance of this protein.

There are two main limitations of the redox array: firstly, a positive signal on the array could also be due to a biotinylated protein that binds to the antibody specific ligand thus resulting in a false positive signal. Therefore, glutathionylated proteins identified using this method should always be independently validated via other methods as shown here for IL-1 sRII and Lyn. Secondly, the protocol is limited by the number and identity of antibodies included on the array and so may be more useful for detecting glutathionylated proteins under specific experimental conditions where a specific subset of proteins are of interest, rather than when a global screening of the entire proteome is required.

Nevertheless, the use of redox arrays could allow a more complete identification of potential targets of redox regulation (redoxome) than conventional proteomics techniques. In fact, this study suggests that 5% of the cellular proteins are susceptible to glutathionylation, while in previous proteomics experiments we never identified more than 40 proteins in the whole proteome^16^, and less than 50 are listed as glutathionylated in the NCBI or UniProt protein databases (http://www.ncbi.nlm.nih.gov/protein?term=%28glutathionylation%29; http://www.uniprot.org/uniprot/?query=keyword:KW-0318). The big advantage of the redox array over more conventional mass spectrometry methods is the much greater sensitivity of this technique. This is of particular importance for defining the redox secretome as opposed to the proteome, given the much lower concentration of secreted proteins compared to intracellular proteins. The ability to quantify the extent of glutathionylation would represent a substantial improvement in this approach, but would require double labelling using two different labels for the protein of interest and the BioGEE, and as such poses significant technical challenges.

The application of this technology to the secretome in disease conditions could represent a new approach in the identification of biomarkers based on their specific redox state. In fact, in some cases the thiol redox state of inflammatory proteins may have better prognostic value than their total protein concentration^30^.

## Methods

Reagents. Lipopolysaccharide (LPS) from E. coli 055:B5, menadione, *N*-Ethylmaleimide (NEM) were all purchased from Sigma Aldrich. Recombinant human IL-1 sRII was purchased from R&D systems.

### Culture and BioGEE treatment of THP-1 cells

THP-1 cells were routinely cultured in T75 tissue culture flasks in RPMI (Sigma) supplemented with 10% fetal bovine serum and 100 U/mL penicillin-streptomycin. For glutathionylation experiments, 30 × 10^6^ cells were washed with RPMI supplemented with 0.5% FBS and 100 U/mL penicillin-streptomycin and resuspended in a total volume of 5 mL of the same medium. BioGEE was added to the culture at a final concentration of 250 μM and incubated at 37 °C for 1 h. Cells were then split into two T75 flasks each containing a total volume of 20 mL of RPMI supplemented with 0.5% FBS and 100 U/mL penicillin-streptomycin and stimulated with 100 ng/mL of LPS for 24 h. Cells and supernatants were then collected by centrifugation. NEM was added to all supernatants and cell lysates to a final concentration of 50 mM immediately after collection.

### Redox array protocol

Membrane-based human antibody arrays (Ray Biotech) were used to detect glutathionylated proteins. These arrays are Human Antibody Arrays consisting of 1000 antibodies immobilised on nitrocellulose membrane (http://www.raybiotech.com/human-l-1000-array-membrane-2.html). Free BioGEE was removed from THP-1 cell lysates and supernatants by eluting from a desalting PD10 column with PBS. Protein concentration was determined by BCA assay (Pierce) and, in the case of cell lysates, adjusted to a concentration of 0.3 mg/mL in blocking buffer (Ray Biotech; proprietary buffer) for application to the antibody array. Cell supernatants were applied to the array without further dilution. After washing, biotinylated proteins were detected according to the manufacturer’s instructions (Ray Biotech) using Streptavidin-HRP and ECL reagent.

### Cloning of human Lyn in pcDNA6

The sequence coding for human Lyn was amplified by PCR from a cDNA clone (MGC; GE Healthcare). The primers used also introduced restriction sites for *Bam*HI and *Xho*I as follows sense: ctag GGATCC ATG GGA TGT ATA AAA TCA AAA GGG and anti-sense: 5′ ctag CTC GAG GC AGG CTG CTG CTG GTA TTG CCC TTC 3′. The resulting fragment was digested with *Bam*HI and *Xho*I and ligated into pcDNA6 (Invitrogen) also digested with *Bam*HI and *Xho*I. The sequence of the construct was verified by sequencing.

### Culture and transient transfection of 293T cells

293T cells were routinely cultured in DMEM (Sigma) supplemented with 10% fetal bovine serum and penicillin and streptomycin. Transient transfections were performed using 25 kDa linear polyethylenimine (PEI) (purchased from Polysciences Inc. PA, USA). Stock solutions of PEI were prepared in water at a concentration of 1 mg/ml, and the pH adjusted to 7.0. The transfection conditions used are essentially those as previously described^31^. Briefly, HEK 293T cells were plated at a density of 0.5 × 10^6^ cells/well in 6-well plates and transfected with 5 μg of DNA. The transfection complex was formed at a DNA:PEI ratio of 1:3 in OPTI-MEM (Life Technologies, Paisley, UK), with a 30 minute incubation at room temperature prior to addition to the cells. 24 h later, culture media were replaced with 1 mL of serum-free DMEM containing 50 ng/mL of recombinant hTNF-α (Peprotech) and cultured for a further 24 h before collecting cells and supernatants. NEM was added to cell lysates to a final concentration of 50 mM immediately after collection to prevent post-lysis glutathionylation by blocking free thiols in both proteins and BioGEE, and also prevent thiol-disulfide exchange reactions that could result in loss of glutathionylation.

### Purification of recombinant Lyn

293T cells expressing recombinant His-tagged Lyn were lysed in cold NP-40 lysis buffer and incubated on ice for 30 min. Cleared supernatants were loaded onto HisTrap columns and allowed to enter the resin by gravity flow. The column was washed with 10 mL of binding buffer, followed by 10 mL of wash buffer, before eluting the bound proteins using 3 mL of elution buffer.

### Cell-free glutathionylation experiments

Fifty ng of recombinant IL-1sRII (R&D systems) or recombinant Lyn purified from HEK293T cells were treated with 100 mM of DTT for 20 min at room temperature to reduce free cysteines. DTT was then removed from the sample on a NAP5 desalting column (GE Healthcare). BioGEE was added to a final concentration of 250 μM and incubated at room temperature for 15 min, followed by the addition of 0.5 mM diamide and a further 15 min incubation at room temperature. The reaction was terminated by the addition of NEM to a final concentration of 50 mM. Free BioGEE was removed by desalting using a NAP5 column as described above. A portion of the sample was reduced with 100 mM DTT (final concentration) to remove the BioGEE label.

### Western blot analysis and immunoprecipitation

Proteins from the cell conditioned media of THP-1 cells were precipitated by the addition of 3 volumes of ice-cold acetone, incubation at −20 °C for 18 hours, and centrifugation at 13,000 × g for 10 min at 4 °C. Protein pellets were solubilized in Laemmli buffer and subjected to Western blot analysis as detailed below. 293T cell culture supernatants were applied to 12% SDS-PAGE gels without prior dilution and transferred to nitrocellulose membranes (Millipore, Watford, UK) by electroblotting at 400 mA, 1.5 h, 4 °C. After blocking in 5% skimmed milk in PBS containing 0.05% Tween 20, membranes were probed with anti-human Lyn monoclonal mouse antibody (Santa Cruz) diluted 1:1000, or anti-human IL-1 sRII mouse antibody, Clone 8.5^32^ followed by detection with anti-mouse-IgG-horseradish peroxidase conjugate diluted at 1:5000 in blocking buffer (Stratagene). In some experiments, glutathionylated proteins were detected by probing membranes with streptavidin-HRP diluted 1:25,000 in 1% BSA. Blots were developed using advanced chemiluminescence (ECL) reagents (GE Healthcare, Amersham, UK) and exposed to autoradiography using Hyperfilm (GE Healthcare). Films were developed using an AGFA Curix 60 developer (Agfa Healthcare, Middlesex, UK). For immunoprecipitations (IP), 5 μg of the corresponding Ab were added to 1.5 mL of cell lysate or 10 mL of cell conditioned media and incubated at 4°C for 18 h on a rotator. One hundred microliters of a 50% slurry of prewashed protein G-agarose beads (GE Healthcare) was then added to each sample, followed by incubation for an additional 4 h at 4°C. The samples were washed four times in PBST, solubilized in Laemmli buffer, and subjected to Western blot analyses as described above.

### ELISA

The concentration of IL-1 sRII was determined in cell supernatants using a commercially available ELISA (R&D systems).

### Mass Spectrometry

For the identification of secreted glutathionylated proteins, desalted supernatants were incubated with 200 μL of streptavidin beads (streptavidin immobilized on agarose CL-4B; Sigma-Aldrich), after which the beads were washed first with PBS and then with NH_4_CO_3_. The beads were then subjected to tryptic digestion, performed with 5 μL of 25 ng/mL trypsin and 45 μL of 25 mM NH_4_ HCO_3_ on ice for 45 min, incubated overnight at 37 °C. The resulting tryptic peptides were extracted by 5% formic acid/50% acetonitrile and concentrated using a SpeedVac (Thermo Scientific).

In a second set of experiments, supernatants from three experiments were pooled and concentrated 120-fold before applying to 500 μL of streptavidin agarose. After extensive washing with PBS and then NH_4_CO_3,_ bound proteins were eluted with 100 mM DTT, concentrated 5-fold and applied to 12% SDS-PAGE gels. The resulting gel bands were excised and subjected to trypsin in-gel digestion essentially as previously described^33^.

After digestion, peptides were dried on SpeedVac, resuspended in 25 μL of 3% (v/v) acetonitrile and 0.1% (v/v) formic acid and then analyzed with a nano-LC1200 system coupled to a Q-TOF 6550 mass spectrometer equipped with a nanospray source and an HPLC-chip cube interface (Agilent Technologies). A 34-min linear gradient (3–75% acetonitrile in 0.1% formic acid), at a flow rate of 350 nL/min, was used to separate peptides on polaris-HR-Chip C18 column (150 mm long × 75 μm inner diameter). Full autoMS1 scans from 290 to 1700 *m/z* and autoMS2 from 59 to 1700 *m/z* were recorded. In every cycle, a maximum of 8 precursors sorted by charge state (2+ preferred and single-charged ions excluded) were isolated and fragmented in the collision cell that was automatically adjusted depending on the *m/z*. Active exclusion of these precursors was enabled after 1 spectrum within 1 min, and the absolute threshold for precursor selection was set to 1000 counts (relative threshold 0.001%). For protein identification, MS/MS peak lists were extracted (merge MSn scans with the same precursor at +/− 30 s retention time and +/− 0.05 m/z tolerance) and compared with the protein databases by using the Spectrum Mill MS proteomics workbench (Agilent Technologies, Rev B.04.00.127). The searches were performed with the following specific parameters: enzyme specificity, trypsin; two missed cleavages permitted; no fixed modifications; variable modifications, methionine oxidation, cysteine carbamidomethylation, pyroglutamic acid (N-termQ); monoisotopic; peptide charge, 2+ and 3+; mass tolerance for precursor ions, 20 ppm; mass tolerance for fragment ions, 50 ppm; ESI-QUAD-TOF as instrument; taxonomy, Human; database, UniProtKB/Swiss-Prot release 2012_11. Only significant hits with a false discovery rate (FDR = 1%) for peptides and proteins were considered.

## Additional Information

**How to cite this article**: Mullen, L. *et al.* Development of ‘Redox Arrays’ for identifying novel glutathionylated proteins in the secretome. *Sci. Rep.* **5**, 14630; doi: 10.1038/srep14630 (2015).

## Supplementary Material

Supplementary data

Supplementary dataset 1

## Figures and Tables

**Table 1:** Secreted proteins identified from 3 biological replicates of LPS-treated THP-1 cells by redox array.

Protein	Full name	Genbank	Biol. Rep 1	Biol Rep 2	Biol Rep 3	Lost with DDT
4-1BB/TNFRSF91	Tumor necrosis factor receptor superfamily, member 9	NM_001561	✓			✓
Aldolase A	Aldolase A	NM_000034	✓			
ApoD	Apolipoprotein D	NM_001647	✓		✓	✓
Beta Defensin 4	Beta Defensin 4	NM_004942	✓	✓	✓	✓
Biglycan	Biglycan	NM_001711	✓	✓	✓	✓
BIK	B-cell lymphocyte	NM_001197	✓	✓	✓	
BNIP2	BCL2/adenovirus E1B 19 kDa protein-interacting protein 2	NM_004330	✓	✓	✓	✓
Calbindin	Calbindin	NM_004929	✓	✓	✓	✓
CD79 alpha	CD79a molecule	NM_001783	✓	✓	✓	✓
Contactin-1*	Contactin-1	NM_001843	✓	✓	✓	✓
Creatinine	Creatinine	N/A	✓	✓	✓	
CSH1#	Chorionic somatomammotropin hormone 1	NM_001317	✓	✓		✓
CSK	Tyrosine-protein kinase CSK	NM_004383	✓	✓	✓	
Cystatin B*	Cystatin B	NM_000100	✓	✓	✓	✓
Endostatin	Endostatin; Collagen type 18 contains Endostatin	NM_030582	✓	✓	✓	
EXTL2*,#	Exostosin-like 2	NM_001439		✓		
FABP4	Fatty acid binding protein 4	NM_001442	✓	✓	✓	✓
FOXN3	Forkhead box N3	NM_005197	✓	✓	✓	
FoxO1/FKHR*,#	Forkhead box O1	NM_002015		✓		
GDF1	GDF1 (N-term)	NM_001492		✓	✓	✓
Ghrelin	Ghrelin	NM_016362	✓	✓	✓	✓
Glypican 3	Glypican 3	NM_00484	✓	✓	✓	✓
HP*,#	Haptoglobin	NM_005143		✓		✓
Hepcidin	Hepcidin	NM_021175	✓	✓	✓	
HSP70	Heat shock protein 70	NM_005345	✓	✓	✓	✓
ICAM-2	Intercellular Adhesion Molecule 2	NM_000873	✓	✓	✓	
L-1 RII*	L-1 Receptor II	NM_004633		✓		✓
L-1 F5/FIL1delta#	Human Interleukin 1 family member 5	NM_173170	✓			✓
Integrin alpha V#	Integrin alpha V	NM_002210	✓	✓		✓
Itk#	L-2-inducible T cell kinase	NM_005546	✓	✓	✓	✓
ITM2B#	Integral membrane protein 2B	NM_021999	✓	✓	✓	✓
LIN41*,#	lineage41	NM_001039111		✓		
Livin	Livin	NM_139317	✓	✓	✓	✓
NELL2#	Neural epidermal growth factor-like 2	NM_006159	✓	✓		✓
Neuropeptide Y#	Neuropeptide Y	NM_000905		✓		✓
NRG2*,#	Pro-neuregulin-2, Neuregulin-2, Pro-NRG2	NM_013981		✓		
Omentin/Intelectin	Omentin/Intelectin	NM_017625.2	✓	✓	✓	✓
PTHLP*,#	Parathyroid Hormone-Like Protein	NM_198965		✓		
RELM alpha/RELM beta	Resistin-like alpha - Renamed RelmB in humans	NM_032579	✓	✓	✓	✓
S100A10	S100A10, ANX2LG, CAL1L, CLP11	NM_002966	✓	✓	✓	✓
S100A4	S100 calcium-binding protein A4	NM_002961	✓	✓	✓	✓
S100A8*,#	S100 calcium-binding protein A8	NM_002964		✓		✓
SART1*,#	Squamous cell carcinoma antigen recognized by T-cells 1	NM_005146		✓	✓	✓
SART3#	Squamous cell carcinoma antigen recognized by T-cells 3	NM_014706		✓		✓
SRMS#	Src-Related Kinase	NM_080823		✓		✓
TFPI*,#	Tissue factor pathway inhibitor	NM_006287	✓	✓	✓	
TGF-beta 5	Transform ing growth factor-beta-5		✓	✓	✓	✓
Thrombopoietin (TPO)*	TSF TPO	NM_000460		✓		
TRAIL R3 (TNFRSF10C)*	TRAIL R3 (TNFRSF10C)	NM_003841		✓	✓	✓
TRPC1*	Transient receptor potential cation channel, subfamily C	NM_003304		✓	✓	
TXK#	Tyrosine-protein kinase TXK	NM_003328		✓		✓
Tyk2#	Tyrosine kinase 2	NM_003331		✓		✓
VDUP-1	Vitamin D3 up-regulated protein 1	NM_006472	✓	✓		
VEGF-C*,#	VEGF-2 is the same as VEGF-C	NM_005429			✓	✓
Vitamin D Receptor	Vitamin D Receptor	NM_000376		✓	✓	✓

^*^indicates proteins identified only in cell supernatants, but not in cell lysates.

^#^indicates proteins identified in THP-1 cell supernatants treated with LPS, but not in supernatants from untreated THP-1 cells.

**Table 2:** Intracellular proteins identified from two biological replicates of LPS-treated THP-1 cells by redox array.

Protein	Full name	Genbank	Biol. Rep 1	Biol Rep 2	Lost with DDT	Known
4-1BB/TNFRSF9	Tumor necrosis factor receptor superfamily, member 9	NM_001561	✓	✓	✓	
Activin B*	Activin B	NM_002193	✓	✓		
Aldolase A	Aldolase A	NM_000034	✓	✓		✓
ApoD	Apolipoprotein D	NM_001647	✓	✓	✓	
Beta Defensin 4	Beta Defensin 4	NM_004942	✓	✓	✓	
Biglycan	Biglycan	NM_001711	✓		✓	
BIK	B-cell lymphocyte	NM_001197	✓	✓		
BNIP2	BCL2/adenovirus E1B 19 kDa protein-interacting protein 2	NM_004330	✓	✓	✓	
Calbindin	Calbindin	NM_004929	✓	✓	✓	
CD79 alpha	CD79a molecule	NM_001783	✓	✓	✓	
CD 163	Cluster of Differntiation 163	NM_203416		✓	✓	
Complement factor H*	Complement factor H	NM_000186	✓			
Creatinine	Creatinine	N/A	✓	✓		
CSH1	Chorionic somatomammotropin hormone 1	NM_001317	✓	✓	✓	
CSK	Tyrosine-protein kinase CSK	NM_004383	✓	✓		
Cytokeratin 18*	Cytokeratin 18	NM_199187	✓	✓	✓	
DLL1*	Delta-like protein 1	NM_005618	✓	✓	✓	
Endostatin	Endostatin; Collagen type 18 contains Endostatin	NM_030582	✓	✓		
Endothelin Receptor A	Endothelin Receptor A	NM_001166055	✓	✓		
FABP4	Fatty acid binding protein 4	NM_001442	✓	✓	✓	✓
FOXN3	forkhead box N3	NM_005197	✓	✓		
Galectin-1*	Galectin-1	NM_002305	✓	✓	✓	✓
GDF1	GDF1 (N-term)	NM_001492		✓	✓	
Ghrelin	Ghrelin	NM_016362	✓	✓	✓	
Glypican 3	Glypican 3	NM_00484		✓	✓	
HADHA*	hydroxyacyl-CoA dehydrogenase	NM_000182	✓	✓	✓	✓
HCR/CRAM-A/B*	Human Chemokine Receptor CRAM-A isoform	NM_019052	✓	✓		
Hepcidin	Hepcidin	NM_021175	✓	✓		
HSP70	Heat shock protein 70	NM_005345	✓	✓	✓	✓
ICAM-2	Intercellular Adhesion Molecule 2	NM_000873	✓	✓		
IL-11*	Interleukin 11	NM_000641	✓	✓	✓	
IL-1 F5/FIL1delta	Human Interleukin 1 family member 5	NM_173170	✓	✓	✓	
IL-17R*	Interleukin 17 receptor A	NM_014339		✓	✓	
Integrin alpha V	Integrin alpha V	NM_002210	✓	✓	✓	
Itk	IL-2-inducible T cell kinase	NM_005546	✓	✓	✓	
ITM2B	Integral membrane protein 2B	NM_021999	✓	✓	✓	
LBP*	Human lipopolysaccharide-binding protein	NM 004139	✓	✓	✓	
LIF*	Leukemia Inhibitory Factor	NM_002309		✓	✓	
LIGHT/TNFSF14*	hHomologous to lymphotoxins	NM_172014		✓	✓	
Lep*	LEPTIN	NM_000230	✓			
Lipocalin-2*	Lipocalin 2	NM_005564	✓	✓	✓	
Livin	Livin	NM_139317	✓	✓	✓	
Lyn*	v-yes-1 Yamaguchi sarcoma viral related oncogene homoloc	NM_002350	✓	✓	✓	
MCSF/CSF1*	Macrophage-colony Stimuating Factor	NM_000757	✓		✓	
NELL2	neural epidermal growth factor-like 2	NM_006159	✓	✓	✓	
Nesfatin*	Nesfatin	NM_005013	✓	✓	✓	
Neuropeptide Y	Neuropeptide Y	NM_000905	✓	✓	✓	
Omentin/Intelectin	Omentin/Intelectin	NM_017625.2	✓		✓	
OPN*	Osteopontin	NM_000582	✓	✓	✓	
p21*	cyclin-dependent kinase inhibitor 1A	NM_078467	✓		✓	
Pancreastatin*	Pancreastatin	NM_001275	✓	✓	✓	
PI 16*	Peptidase Inhibitor 16	NM_153370	✓	✓	✓	
Podocalyxin*	Podocalyxin	NM_001018111	✓	✓	✓	
Prostasin*	Prostasin	NM_002773	✓	✓	✓	
RELMalpha/RELM beta	resistin-like alpha - Renamed RelmB in humans	NM_032579	✓	✓	✓	
S100A10	S100A10, ANX2LG, CAL1L, CLP11	NM_002966	✓	✓		
S100A4	S100 calcium-binding protein A4	NM_002961	✓	✓	✓	✓
S-100b*	S100 calcium binding protein B	NM_006272	✓	✓	✓	✓
SART3	squamous cell carcinoma antigen recognized by T-cells 3	NM_014706	✓	✓	✓	
SCG3*	Secretogranin III	NM_013243	✓	✓	✓	
SLPI	antileukoproteinase (abbr.ALP)	NM_003064	✓	✓		
SMAC	second mitochondria-derived activator of caspase	NM_019887	✓	✓		
SRMS	Src-Related Kinase	NM_080823	✓	✓	✓	
SSEA-1*	Stage-Specific Embryonic Antigen1, CD15	NM_002033	✓	✓	✓	
TGF-beta 5	transforming growth factor-beta-5		✓	✓		
BDCA-3*	Thrombomodulin	NM_000361	✓	✓	✓	
TNFSF3*	Lymphotoxin beta	NM_009588	✓	✓	✓	
TNK1*	tyrosine kinase, non-receptor, 1	NM_003985	✓		✓	
Troponin C*	troponin C type 1	NM_003280	✓	✓		
TXK	tyrosine-protein kinase TXK	NM_003328	✓	✓	✓	
Tyk2	tyrosine kinase 2	NM_003331	✓	✓	✓	
uPA*	Uurokinase plasminogen activator inducing activity	NM_002658	✓		✓	
VDUP-1	vitamin D3 up-regulated protein 1	NM_006472	✓	✓		
Vitamin D Receptor	Vitamin D Receptor	NM_000376	✓	✓	✓	

^*^Indicates proteins identified only in cell lysates, but not in cell supernatants.

**Table 3:** Secreted proteins identified from two biological replicates of untreated THP-1 cells by redox array.

Protein	Full name	Genbank	Biol. Rep 1	Biol Rep 2	Lost with DDT
4-1BB/TNFRSF9	Tumor necrosis factor receptor superfamily, member 9	NM_001561	✓	✓	✓
Aldolase A	Aldolase A	NM_000034	✓		
ApoD/Apolipoprotein D	Apolipoprotein D	NM_001647	✓	✓	✓
Beta Defensin 4	Beta Defensin 4	NM_004942	✓	✓	✓
Biglycan	Biglycan	NM_001711	✓		✓
BIK	B-cell lymphocyte	NM_001197	✓	✓	✓
BNIP2	BCL2/adenovirus E1B 19 kDa protein-interacting protein 2	NM_004330	✓	✓	
Calbindin	Calbindin	NM_004929	✓	✓	✓
CD 163#	Cluster of Differntiation 163	NM_203416	✓	✓	✓
CD79 alpha	CD79a molecule	NM_001783	✓	✓	✓
Contactin-1*	Contactin-1	NM_001843	✓	✓	✓
Creatinine	Creatinine	N/A	✓	✓	
CSK	Tyrosine-protein kinase CSK	NM_004383	✓	✓	
Cystatin B*	Cystatin B	NM_000100	✓	✓	✓
Endostatin	Endostatin; Collagen type 18 contains Endostatin	NM_030582	✓	✓	
Endothelin Receptor A#	Endothelin Receptor A	NM_001166055	✓		
FABP4	Fatty acid binding protein 4	NM_001442		✓	✓
FOXN3	Forkhead box N3	NM_005197	✓	✓	
GDF1	GDF1 (N-term)	NM_001492		✓	✓
Ghrelin	Ghrelin	NM_016362	✓	✓	✓
Glypican 3	Glypican 3	NM_00484		✓	✓
Hepcidin	Hepcidin	NM_021175		✓	
HSP70	Heat shock protein 70	NM_005345	✓	✓	✓
ICAM-2	Intercellular Adhesion Molecule 2	NM_000873	✓	✓	
IL-1 RII*	IL-1 Receptor II	NM_004633	✓		✓
IL-1 R4/ST2*,#	IL-1 R4/ST2	NM_016232		✓	✓
Livin	Livin	NM_139317	✓	✓	✓
Omentin/Intelectin	Omentin/Intelectin	NM_017625.2	✓	✓	✓
RELM alpha/RELM beta	Resistin-like alpha - Renamed RelmB in humans	NM_032579	✓	✓	✓
ROBO4*,#	Human Roundabout homolog 4	NM_019055	✓	✓	✓
S100A10	S100A10, ANX2LG, CAL1L, CLP11	NM_002966	✓	✓	
S100A4	S100 calcium-binding protein A4	NM_002961	✓	✓	✓
SLPI#	Antileukoproteinase (abbr.ALP)	NM_003064		✓	✓
SMAC#	Ssecond mitochondria-derived activator of caspase	NM_019887	✓		
TGF-alpha*,#	TGF-alpha. (transformed cell growth factor)	NM_003236	✓		
TGF-beta 5	Transforming growth factor-beta-5		✓	✓	
TRAIL R3 (TNFRSF10C)'	TRAIL R3 (TNFRSF10C)	NM_003841		✓	✓
TRPC1*	Transient receptor potential cation channel, subfamily C	NM_003304		✓	
VDUP-1	Vitamin D3 up-regulated protein 1	NM_006472	✓	✓	
Vitamin D Receptor	Vitamin D Receptor	NM_000376	✓		

^*^indicates proteins identified only in cell supernatants, but not in cell lysates.

^#^indicates proteins identified in THP-1 cell supernatants treated with LPS, but not in supernatants from untreated THP-1 cells.
