# Supplementary material for: Development of ‘Redox Arrays’ for identifying novel glutathionylated proteins in the secretome
Source: Sci Rep. 2015 Sep 29;5:14630. doi: 10.1038/srep14630 (PMC4586893; doi:10.1038/srep14630)
Supplement: Supplementary data [file srep14630-s1.pdf]

Development of 'Redox Arrays' for identifying novel glutathionylated proteins in the secretome

[1]Lisa Mullen*, [1]Miles Seavill, [1]Raneem Hammouz, [4]Barbara Bottazzi, [2,3]Phillippe Chan, [2,3]David Vaudry, [1]Pietro Ghezzi

[1]Brighton and Sussex Medical School, Falmer, Brighton BN1 9RY

[2]Institute for Research and Innovation in Biomedicine, Rouen University, 76821 Rouen, France

[3]Platform in Proteomics PISSARO, 76821 Mont-Saint-Aignan, France

[4] Humanitas Clinical & Research Center Via Manzoni, 113, 20089 Rozzano, Milano, Italy

*****Corresponding author. Email: l.mullen@bsms.ac.uk

Supplementary methods.

Recombinant TNF (Peprotech, London, UK) or Prdx2 purified from transiently transfected 293T cells were tested for in vitro glutathionylation as described in the main Methods section.

Supplementary Figure Legends

Supplementary Figure 1. TNF is not glutathionylated in vitro. Recombinant TNF or Prdx2 (as a positive control) were incubated with BioGEE and diamide, before analyses by Western blotting. Membranes were probed with streptavidin-HRP to detect the presence of the BioGEE label or anti-TNF antibody to confirm the presence of the protein.

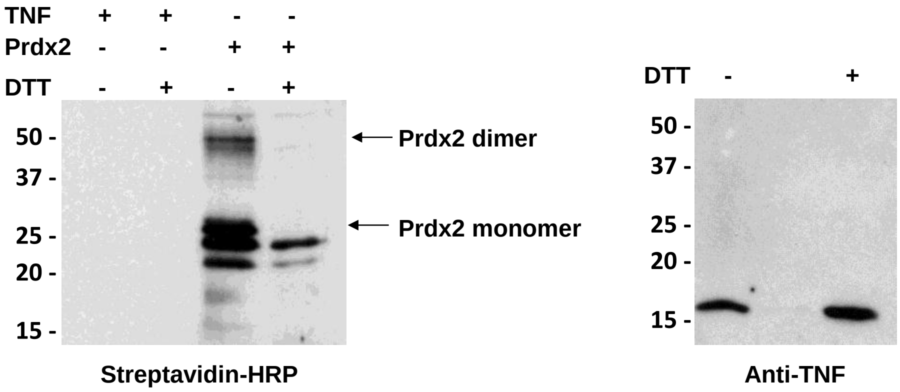

Supplementary Figure 1
